# 

**Published:** 1896-01-04

**Authors:** 


### CONTENTS.

Annus Medicus, 1895 - 225

The Relation between Hæmic and Organic Cardiac Murmurs - 228

Notes and News - 230

Medical Progress and Hospital Clinics—

PROGRESS IN MEDICINE.—Diseases of the Lungs and Pleuræ - 231

Diseases of the Pancreas - 232

PROGRESS IN SURGERY.—Movable Kidney - 233

Surgery of the Nerves - 234

NEW APPLIANCES AND THINGS MEDICAL - 235

Annotations.—The Prevention of Epidemics—Why Hospital Saturday Fails in London—Sanitation for Fishes, and Mayors - 229

Editor's Letter-Box - 236

The Institutional Workshop—

HOSPITAL COMMITTEES AND HOSPITAL SECRETARIES - 237

THE STORY OF THE INSANE FROM YEAR TO YEAR - 237

HOSPITAL MEETINGS - 238

CONSTRUCTION NOTES - 239

Scraps and Gleanings - 240

The Book World of Medicine and Science - 241

### EXTRA SUPPLEMENT.—THE NURSING MIRROR.

News from the Nursing World.—From Year to Year—The Corney Grain Cot—Royal Free Students and Nurses—Earnings of Private Nurses—Nurses at Wotton-under-Edge—A Dublin Training School—Nuns as Nurses—Queen's Nurses in Scotland —Nursing in Russia—Sisters in Hong-Kong—Home Comforts in the Colonies—Prince Alfred Hospital, Sydney—District Nursing in Melbourne — Our Needlework Competition—The Royal National Pension Fund for Nurses—Short Items - cix
Lectures on Nursing. III.—Dust, Dusting, and Disinfection - cxi
Food, with Special Relation to the Sick. II. - cxii
Practical Aspect of a Nurse's Life - cxiii
Christmas Entertainments - cxiv
Appointments - cxvi
Novelties for Nurses - cxvi
Presentation - cxv
Notes and Queries - cxv
Christmas in the Provinces - cxv
French Schools for Trained Nurses: Their Origin and Organisation - cxvi
The Princess Maud Marriage Present Fund - cxvi

